# Haga usted el diagnóstico. Primera parte

**DOI:** 10.7705/biomedica.6977

**Published:** 2023-08-30

**Authors:** Yenny Ariza, Cristian Leonardo Cubides, Daniel Alejandro Cubillos, Carmen Lucía Roa, José Camilo Álvarez, Sonia Isabel Cuervo-Maldonado

**Affiliations:** 1 Grupo de Medicina Interna e Infectología, Instituto Nacional de Cancerología, Bogotá, D.C., Colombia Grupo de Medicina Interna e Infectología Instituto Nacional de Cancerología Bogotá, D.C. Colombia; 2 Grupo de Investigación en Enfermedades Infecciosas en Cáncer y Alteraciones Hematológicas, Instituto Nacional de Cancerología, Bogotá, D.C., Colombia Grupo de Investigación en Enfermedades Infecciosas en Cáncer y Alteraciones Hematológicas Instituto Nacional de Cancerología Bogotá, D.C. Colombia; 3 Facultad de Medicina, Universidad Nacional de Colombia, Bogotá, D.C., Colombia Universidad Nacional de Colombia Facultad de Medicina Universidad Nacional de Colombia Bogotá, D.C. Colombia

Se trata de un hombre de 23 años, natural de Cantaura (Venezuela) y procedente de Yopal (Casanare), obrero de construcción. Como antecedentes de importancia, refirió haber tenido malaria cuatro años antes, diagnosticada y tratada en Venezuela; además, fumó un paquete de cigarrillos al día durante dos años (índice paquetes-años, IPA=2).

Consultó inicialmente al Hospital de la Orinoquia por presentar dolor de 4 meses de evolución en la región lumbosacra derecha, irradiado al miembro inferior derecho, acompañado de fiebre no cuantificada, aparición de masas en la región cervical e hiporexia. No refirió síntomas respiratorios, gastrointestinales, ni lesiones mucocutáneas.

Por la presencia de adenopatías cervicales y axilares, se practicó una biopsia ganglionar que informó: “Ganglio linfático alterado por presencia de granuloma con abundantes eosinófilos y estructuras redondeadas intracelulares que sugieren hongos”. A pesar de este hallazgo y con sospecha de linfoma no Hodgkin, se remitió para confirmación de la neoplasia hematológica maligna y tratamiento.

Al ingreso a la institución, se encontró un paciente en regular estado general, consciente, alerta y orientado, con tensión arterial de 102/62 mm Hg; frecuencia cardiaca de 80 latidos por minuto; frecuencia respiratoria de 18 por minuto; temperatura de 36,2 °C; saturación de oxígeno de 90 % al medio ambiente, peso de 40 kg; talla de 162 cm; índice de masa corporal de 15,24.

**Figura 1:**
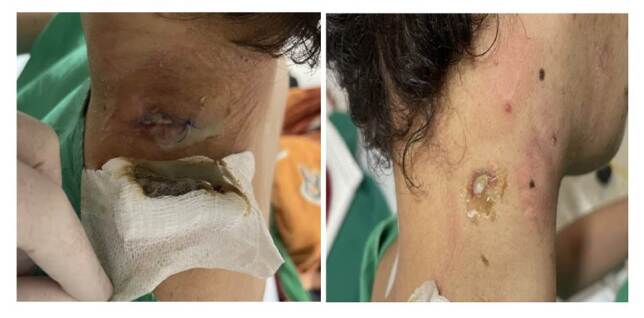
Adenopatías cervicales bilaterales, algunas con ulceración y salida de secreción purulenta

La cavidad oral se encontraba sin lesiones en mucosa yugal, encías, ni lengua. Había múltiples adenopatías cervicales y axilares, localizadas en estación Ia, Ib, II y V; la de mayor compromiso se encontró en la región cervical derecha, de 5 cm, aproximadamente, ulcerada y con secreción hematopurulenta (figura 1).

El examen cardiopulmonar fue normal; en el abdomen, se palpaba el borde hepático por debajo del reborde costal y el bazo se pudo percutir. No había lesiones en la piel. En el examen neurológico, el paciente estaba consciente, alerta, orientado en las tres esferas y sin déficit sensitivo, ni motor.

Con el diagnóstico de linfadenitis cervical y axilar con abscesos, se inició tratamiento con ampicilina-sulbactam y, dado que no hubo mejoría alguna, se adicionó vancomicina durante 10 días, sin que se observara ninguna modificación clínica.

En el cuadro hemático de ingreso, se reportó: leucocitosis (12.110 cél/μl), neutrofilia (7.120 cél/μl), linfocitos normales (1.840 cél/μl), anemia moderada (hemoglobina = 8,41 g/dl), normocítica, normocrómica, heterogénea, plaquetas normales (384.000 cél/μl). La química sanguínea estaba dentro de rangos normales (sodio = 138 mmol/L; potasio = 4,5 mmol/L; magnesio = 1,7 mmol/L; fósforo = 5,5 mg/dl); la función renal era normal (creatinina = 0,8 mg/ dl; BUN = 13 mg/dl); PCR de 7,6 mg/dl; ELISA para HIV, no reactiva.

**Figura 2:**
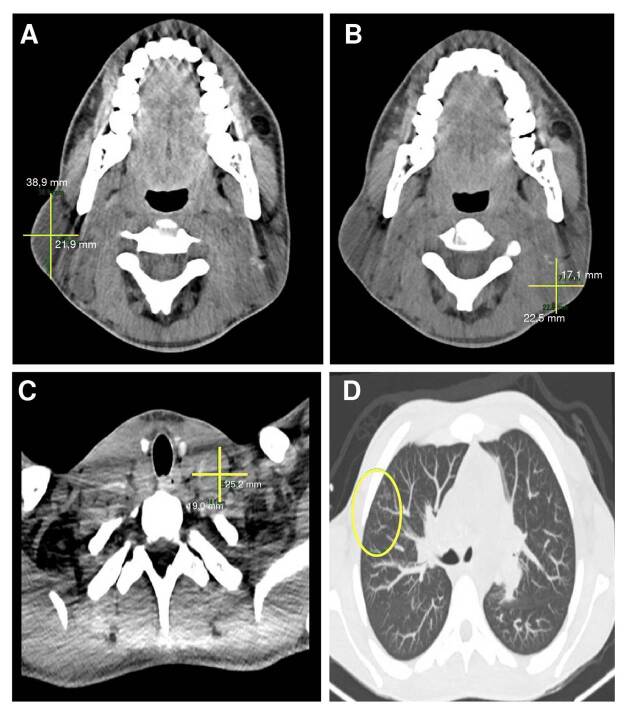
A y B. Tomografía de cuello. Se visualizan adenomegalias en todas las estaciones ganglionares cervicales, algunas con calcificaciones centrales. C y D. Tomografía de tórax. Adenomegalias en todas las estaciones mediastinales axilares bilaterales y supraclaviculares especialmente izquierdas, algunas con calcificaciones centrales. Escasas opacidades centrolobulillares de tipo "árbol en gemación" en el segmento anterior del lóbulo superior derecho.

En las tomografías, se apreciaron adenomegalias en todas las estaciones ganglionares cervicales, algunas con calcificaciones centrales; en el tórax, adenomegalias en todas las estaciones mediastinales axilares bilaterales y supraclaviculares, especialmente las izquierdas, algunas con calcificaciones centrales; asimismo, escasas opacidades centrolobulillares de tipo “árbol en gemación” en el segmento anterior del lóbulo superior derecho (figura 2); en el abdomen y en la pelvis, conglomerados ganglionares y adenomegalias retroperitoneales, en cadenas ilíacas externas e inguinales bilaterales, algunos con calcificaciones centrales y hepatoesplenomegalia.

Durante la estancia hospitalaria, el paciente no manifestó nuevos síntomas y con el tratamiento antimicrobiano inicial no hubo modificación de los síntomas al ingreso. El resultado de la segunda biopsia del ganglio axilar permitió dirigir el tratamiento y el paciente evolucionó hacia la mejoría.

**Preguntas**

1. ¿Cuál es su diagnóstico?
2. Basado en su diagnóstico, ¿cuál es el tratamiento indicado?

